# Mapping the network structure of self-reported everyday executive-functioning difficulties in secondary-school and university student samples from Honduras

**DOI:** 10.3389/fpsyg.2026.1918948

**Published:** 2026-09-23

**Authors:** Marcio Alexander Castillo-Díaz, Dulce Stephania Campos Guillén, Ivette Carolina Rivera Zelaya, Carlos Alberto Henao Periañez

**Affiliations:** 1 Graduate Program in Psychometrics and Educational Evaluation and Department of Psychology, Faculty of Social Sciences, Universidad Nacional Autónoma de Honduras, Tegucigalpa, Honduras; 2 Department of Pedagogy, Campus Cortés, Universidad Nacional Autónoma de Honduras, San Pedro Sula, Honduras; 3 Doctoral Program in Psychology and Department of Psychology, Faculty of Social Sciences, Universidad Nacional Autónoma de Honduras, Tegucigalpa, Honduras; 4 School of Nursing, Faculty of Health, Universidad del Valle, Cali, Colombia

**Keywords:** executive functions, higher education, learning regulation, network analysis, secondary education, self-reported executive functioning

## Abstract

**Background:**

Executive functions support students’ ability to regulate cognition, behavior, and emotion in response to academic demands, thereby providing a regulatory foundation for learning. However, little is known about how self-reported everyday executive-functioning difficulties are conditionally related, particularly in Central American educational settings. This study mapped the network structure of self-reported everyday executive-functioning difficulties in secondary-school and university student samples from Honduras.

**Methods:**

A cross-sectional study was conducted with 2,330 students, including 810 secondary-school students and 1,520 university students. Executive functioning was assessed using the Executive Functioning Questionnaire (EFECO), an ecological self-report measure. Gaussian graphical models were estimated to examine the global network, node centrality, predictability, accuracy, and stability. A secondary exploratory comparison was also conducted between the two independently recruited samples.

**Results:**

The global network was dense, retaining 23 of 28 possible edges and showing high transitivity. The strongest associations were observed between inhibition and emotional control, planning and working memory, and initiative and working memory. Planning, working memory, and initiative had the highest centrality estimates in the global network. These nodes also showed high predictability (*R*^2^ = 0.62–0.68). Centrality stability was high for both strength and expected influence. The exploratory comparison between the secondary-school and university student samples did not detect statistically significant differences in overall network structure or global strength, although localized sample-specific contrasts were observed in selected edges and centrality indices.

**Discussion:**

These findings extend executive-function network research beyond performance-based tasks by characterizing conditional associations among EFECO scores in an underrepresented Central American context. Centrality estimates reflect relative connectivity rather than causal importance, and the nonsignificant global comparison does not establish network equivalence. Given the cross-sectional design, a single self-report instrument, and independently recruited nonprobability samples, longitudinal, multimethod, and multisite studies are needed to establish the stability, generalizability, and educational relevance of these network patterns.

## 1 Introduction

### 1.1 Executive functions and self-regulated learning

Academic success depends not only on what students know, but also on how they regulate learning in response to academic demands. Self-regulated learning (SRL) offers a broad framework for understanding this process, encompassing the coordination of cognitive, metacognitive, behavioral, motivational, and affective dimensions of goal-directed learning (Panadero, 2017; Theobald, 2021; Xu et al., 2023). Students are understood as active agents who plan, monitor, control, and evaluate their learning through cognitive, metacognitive, and resource-management strategies, including attention, effort, time management, and the organization of study conditions (Theobald, 2021; Sáez-Delgado et al., 2023).

Although SRL provides a comprehensive educational account of how students regulate learning, it does not fully explain the cognitive-control capacities that make such regulation possible under demanding academic conditions (Follmer and Sperling, 2016; Rutherford et al., 2018). This gap connects SRL with the literature on executive functions (EFs), which has examined the cognitive processes that support goal-directed control of attention, behavior, and emotion (Diamond, 2013; Roebers, 2017). Recent systematic and scoping reviews indicate that SRL and EFs should be understood as related but non-equivalent constructs, given their low-to-moderate empirical associations, measurement-dependent overlap, and distinct theoretical origins in educational psychology and neuropsychology (Dörrenbächer-Ulrich and Bregulla, 2024; Philpott-Robinson et al., 2026). Accordingly, EFs may be conceptualized as cognitive mechanisms that contribute to academic regulation, without encompassing the broader motivational, affective, behavioral, and contextual scope of SRL (Follmer and Sperling, 2016; Rutherford et al., 2018; Manuhuwa et al., 2023).

Executive functions are commonly defined as a family of top-down control processes that allow individuals to regulate cognition, behavior, and emotion in the service of goal-directed action (Diamond, 2013). The literature generally organizes EF around core processes—inhibitory control, working memory or updating, and cognitive flexibility or shifting—which in turn support more complex abilities such as planning, reasoning, problem solving, and decision making (Miyake et al., 2000; Diamond, 2013; Baggetta and Alexander, 2016). This multidimensional nature has generated ongoing debate about whether EF should be modeled as a unitary control system or as separable but interrelated processes, and whether to distinguish relatively “cool” cognitive control from “hot” executive processes involving affective or motivational demands (Hofmann et al., 2012; Karr et al., 2018). These debates make measurement especially consequential, because different approaches may capture different aspects of executive functioning and lead to different interpretations of its organization (Pitta, 2026).

### 1.2 Everyday executive-function assessment and network analysis

Executive functions have been assessed through multiple approaches, most commonly performance-based tasks and self- or informant-report rating scales (Chan et al., 2008; Nyongesa et al., 2019; Souissi et al., 2022). Performance-based tasks estimate executive efficiency under standardized conditions, whereas rating scales assess perceived executive difficulties as they appear in everyday contexts (Chan et al., 2008; Isquith et al., 2013; Toplak et al., 2013). These approaches should not be treated as interchangeable. Performance-based tasks offer experimental control, but they are affected by task impurity, limited ecological correspondence, and a focus on optimal performance under high structure rather than typical behavior in daily life (Toplak et al., 2013; Nyongesa et al., 2019; Pitta, 2026).

Rating scales, in turn, may be influenced by self-perception and rater bias, but they provide information about how executive difficulties are experienced in daily academic life (Isquith et al., 2013; Soto et al., 2020; Manuhuwa et al., 2024). This distinction is relevant because performance-based and rating-scale measures appear to capture related but different aspects of executive functioning, as reflected in their limited convergence (Toplak et al., 2013). Self-report instruments are therefore particularly relevant when the aim is to examine students’ everyday executive regulation in educational settings rather than optimal cognitive performance under laboratory-like conditions (Isquith et al., 2013; Soto et al., 2020; Manuhuwa et al., 2024).

From this rating-scale perspective, the Executive Functioning Questionnaire (EFECO) follows an ecological-operational logic similar to that of widely used executive-function rating scales such as the Behavior Rating Inventory of Executive Function (BRIEF), by assessing perceived difficulties across everyday executive domains (Gómez, 2015; Ramos-Galarza et al., 2019; Barrios-Fernandez et al., 2021). Previous psychometric studies have examined the EFECO through latent models, supporting its broad factorial organization and showing that its domains are conceptually distinguishable but substantially interrelated (Ramos-Galarza et al., 2019). However, latent models do not directly describe how specific executive domains are conditionally connected to one another or which domains occupy more central positions within a domain-level network. This requires an analytic approach capable of modeling executive-function domains as mutually connected components rather than only as indicators of an underlying construct.

In network analysis, psychological and cognitive phenomena are examined as systems of interrelated components, in which variables are represented as nodes and their associations as edges (Van Der Maas et al., 2017; Hevey, 2018). Within this framework, and unlike conventional bivariate correlations, a Gaussian graphical model estimates conditional associations between pairs of domains after controlling for all remaining domains (Epskamp and Fried, 2018; Borsboom et al., 2021). This approach is useful for constructs such as EF, whose components are theoretically distinguishable but empirically interconnected, and for which single tasks or domains may reflect multiple regulatory processes (Karr et al., 2022; Younger et al., 2023). Network models allow researchers to examine centrality, edge strength, community structure, predictability, and overall topology, thereby complementing rather than replacing latent-variable approaches that focus primarily on shared variance (Guyon et al., 2017; Haslbeck and Waldorp, 2018; Borsboom et al., 2021).

Empirical network studies have begun to clarify how EF domains are organized across development. In a life-span sample, inhibition, shifting, and updating/working memory differentiated from childhood to adolescence and dedifferentiated during adulthood, with shifting emerging as a central component from preadolescence onward (Karr et al., 2022). In children and adolescents, EF networks also showed age- and training-related changes, with children’s post-training networks becoming more similar to adolescent networks, although these findings were described as preliminary (Menu et al., 2022). Using an accelerated longitudinal design, EF tasks were organized into three stable components by age 10, while the composition of those components continued to refine through at least age 14 (Younger et al., 2023).

Network evidence also suggests that the organization of EF domains may vary across populations and analytic approaches. In children and adolescents with and without ADHD, comparable mean EF test performance coexisted with different network parameters, including lower shifting centrality and weaker relations among EF components in the ADHD group (Karr et al., 2024). Across ages 7.8–15.3 years, structural equation modeling, network modeling, and latent network modeling converged in supporting EF differentiation, while network models revealed task connections not captured by latent models (Menu et al., 2024). In undergraduates, however, neither factor analysis nor network analysis produced a fully compelling higher-order grouping of EF tasks, reinforcing the need to treat network models as complementary rather than definitive tools for studying EF organization (Holmén et al., 2024).

### 1.3 Developmental and educational contexts of executive functioning

Secondary-school and university students are situated at different but intertwined points of the developmental and educational trajectory relevant to executive functioning. During adolescence—the period that characterizes most secondary students—executive functions continue to mature, though not uniformly across components (Huizinga and Smidts, 2010; Theodoraki et al., 2020; Tervo-Clemmens et al., 2023). Large-scale evidence suggests that EF performance shows rapid development from late childhood to mid-adolescence, smaller but significant changes from mid- to late adolescence, and stabilization toward adult levels around 18–20 years of age (Tervo-Clemmens et al., 2023). Inhibition, working memory, shifting, and hot/cool executive processes may follow different developmental trajectories during this period (Poon, 2018; Theodoraki et al., 2020), and teacher-rated executive functioning has been shown to predict current and later achievement across middle and secondary school grades (Samuels et al., 2016).

Alongside age-related maturation, higher education presents academic demands that differ from those of secondary school. University learning requires greater autonomy, reduced external guidance, management of multiple deadlines, and more independent planning, monitoring, and evaluation of academic work (Baars et al., 2015; Vosniadou, 2020). Evidence also indicates that self-reported attention, planning, self-control, and self-monitoring predict study progress in first-year students (Baars et al., 2015), while systematic review evidence suggests that the secondary-to-tertiary transition involves changes in cognitive and metacognitive regulation (Dörrenbächer-Ulrich et al., 2024). Together, these bodies of evidence make both developmental maturation and educational context plausible sources of variation in executive functioning.

To our knowledge, no directly comparable studies were identified in the executive-function network literature. Nevertheless, cross-sectional comparisons between secondary-school and university samples have been reported for executive functioning assessed with performance-based tasks (Marquez-Ramos et al., 2023) and for regulatory processes closely related to everyday executive functioning. The latter studies examined affective, behavioral, and cognitive forms of regulation (Ben-Eliyahu and Linnenbrink-Garcia, 2015), motivational orientations and cognitive, metacognitive, and resource-management learning strategies (Hederich-Martínez et al., 2018), and metacognitive awareness through confidence judgments and calibration accuracy (Nederhand et al., 2021). Such comparisons are descriptively informative for characterizing common and sample-specific patterns. However, they cannot establish developmental change or isolate the contribution of educational level because age, selection into higher education, and task or examination characteristics may covary with group membership (Hederich-Martínez et al., 2018; Nederhand et al., 2021).

### 1.4 Knowledge gaps and study rationale

Despite the arguments developed above, several knowledge gaps persist. First, at the methodological level, existing EF network studies have relied on performance-based tasks (Karr et al., 2022, 2024; Menu et al., 2022, 2024; Younger et al., 2023; Holmén et al., 2024), leaving unclear whether their findings generalize to self-reported executive functioning assessed through ecological rating scales such as the EFECO. This distinction is important because performance-based and rating-scale approaches show limited convergence and capture different aspects of executive functioning (Toplak et al., 2013). Second, at the geographic level, EF research remains concentrated in samples from Western, educated, industrialized, rich, and democratic (WEIRD) societies, with fewer contributions from Latin America and especially Central America (Gago-Galvagno et al., 2024; Laurence, 2026). In Honduras, available EF evidence is limited, and ecological self-report approaches remain underexamined. To our knowledge, the only available evidence on EF in Honduras comes from performance-based assessments in pediatric samples—specifically, a prevalence study of low scores on executive-function tasks in children aged 6–14 years (Benito-Sánchez et al., 2020). Third, as a secondary comparative gap, it remains unknown whether the network structure of self-reported everyday executive functioning shows common and sample-specific features in secondary-school and university student populations. This comparison remains educationally relevant because these populations face different developmental and academic conditions (Tervo-Clemmens et al., 2023; Dörrenbächer-Ulrich et al., 2024).

Accordingly, the primary aim of this study was to map the network structure of self-reported everyday executive-functioning difficulties in a large, combined sample of secondary-school and university students from Honduras. Specifically, it sought to identify the most central executive-function domains and examine the relational organization of everyday cognitive, behavioral, and emotional regulation. After establishing item-level measurement invariance, a secondary exploratory aim was to examine whether the estimated network parameters showed common and sample-specific features across the two independently recruited samples. These contrasts were not intended to estimate developmental change or an effect attributable uniquely to educational level. By integrating an ecological model of executive functioning with network analysis in a Latin American low- and middle-income context, this study contributes primarily methodological and geographically situated evidence, while the exploratory comparison provides hypothesis-generating evidence on between-sample variation.

## 2 Method

### 2.1 Setting and participants

Data were collected from two educational settings in Honduras representing different stages of the educational trajectory: secondary education and higher education. The secondary education sample was recruited from two public educational institutions in northern Honduras, whereas the university sample was drawn from a public university campus. To preserve institutional confidentiality, the names of the participating institutions are not reported.

Two independent samples were included. The first sample, corresponding to secondary school students, was drawn from a previous study (Menjivar Alas et al., 2026). It comprised adolescents enrolled in lower and upper secondary education. According to institutional records reported in that study, the two participating schools had a combined enrollment of 3,320 students, which constituted the target population. In the Honduran educational system, lower secondary education corresponds to Grades 7–9, whereas upper secondary education corresponds to Grades 10–12. The second sample comprised undergraduate students enrolled at the participating university campus during the first academic period of 2023. Institutional records indicated a total undergraduate enrollment of 44,227 students, which constituted the target population for this group.

For the secondary school sample, eligible participants were students aged 11–17 years who were officially enrolled in lower or upper secondary education at the participating institutions and who provided assent, together with written informed consent from a parent or legal guardian. Students were excluded if they declined participation, if assent or parental consent was not obtained, if they were absent during data collection, or if their questionnaires contained substantial missing information that prevented reliable analysis.

For the university sample, eligible participants were undergraduate students aged 18–25 years who were officially enrolled at the participating university campus and provided informed consent prior to participation. Students enrolled exclusively in postgraduate or non-degree programs, those who declined participation, and those with substantial missing data preventing calculation of the study variables were excluded.

Participants were recruited using non-probability purposive sampling. Before analysis, questionnaires from both samples were screened for completeness and invariant response patterns. Only participants with complete responses to all 67 EFECO items were retained. Questionnaires presenting the maximum possible long-string pattern—operationalized in this study as selecting the same response option across all 67 items—were also excluded (Curran, 2016). Together, these screening criteria excluded 17 of 827 secondary-school participants (2.1%) and 29 of 1,549 university participants (1.9%). Given the small proportion of exclusions and the need to estimate all domain scores and network associations from the same observations, complete-case analysis was used and no imputation was performed.

The final analytic sample included 2,330 students: 810 secondary-school students and 1,520 university students. The secondary education sample represented 24.4% of its target population (810/3,320), whereas the university sample represented 3.4% of its target population (1,520/44,227). The full sample comprised 1,253 female (53.8%) and 1,077 male (46.2%) participants, aged 11–25 years (*M* = 18.41, SD = 3.29). Sample-specific sociodemographic and educational characteristics are presented in Table 1.

**Table 1:** Sociodemographic and educational characteristics of the secondary-school and university samples (*N* = 2,330).

Characteristic	Secondary school students (*n* = 810)	University students (*n* = 1,520)
Age, years, mean ± SD (range)	14.78 ± 1.72 (11–17)	20.35 ± 2.07 (18–25)
Sex, *n* (%)		
Male	450 (55.6)	627 (41.3)
Female	360 (44.4)	893 (58.7)
Educational level, *n* (%)		
Lower secondary education (grades 7–9)	427 (52.7)	–
Upper secondary education (Grades 10–12)	383 (47.3)	–
Field of study, *n* (%)		
Biological and health sciences	–	290 (19.1)
Economic and administrative sciences	–	667 (43.9)
Engineering and physical–mathematical sciences	–	230 (15.1)
Social sciences, humanities, and arts	–	333 (21.9)

*M*, mean; SD, standard deviation.

### 2.2 Measuring instrument

#### 2.2.1 Executive functioning

Executive functioning was assessed using the Executive Functioning Questionnaire (EFECO), an ecological self-report measure designed to evaluate executive functioning through behavioral manifestations in everyday contexts (Gómez, 2015; Ramos-Galarza et al., 2019). The original instrument was developed as an informant-report measure, whereas the self-report version used in this study includes 67 items rated on a four-point Likert-type scale ranging from 0 (never) to 3 (very often), with higher scores indicating greater perceived executive-functioning difficulties (Ramos-Galarza et al., 2019). This version assesses eight executive-functioning dimensions: inhibition, flexibility, emotional control, monitoring, planning, organization, initiative, and working memory. Previous research has provided evidence of validity and reliability for the EFECO in the Honduran context (Menjivar Alas et al., 2026).

For the present study, evidence from the full sample supported the adequacy of the eight-factor correlated model, *χ*^2^ (2,116) = 12,651.19, CFI = 0.975, TLI = 0.974, RMSEA = 0.046, 90% CI [0.045, 0.047], and SRMR = 0.055. Standardized factor loadings were generally adequate across dimensions: Working memory (*λ* = 0.520–0.808), Planning (*λ* = 0.499–0.713), Organization (*λ* = 0.574–0.804), Monitoring (*λ* = 0.414–0.663), Initiative (*λ* = 0.368–0.732), Inhibition (*λ* = 0.498–0.705), Flexibility (*λ* = 0.461–0.616), and Emotional control (*λ* = 0.670–0.778). Reliability estimates were adequate to high across most dimensions, as indicated by McDonald’s omega (*ω*) and composite reliability (CR): Working memory (*ω* = 0.864, CR = 0.902), Planning (*ω* = 0.779, CR = 0.831), Organization (*ω* = 0.835, CR = 0.887), Monitoring (*ω* = 0.741, CR = 0.808), Initiative (*ω* = 0.806, CR = 0.863), Inhibition (*ω* = 0.765, CR = 0.830), Flexibility (*ω* = 0.658, CR = 0.731), and Emotional control (*ω* = 0.847, CR = 0.884). Overall, these results supported the adequacy of the eight EFECO dimensions for use as indicators of self-reported executive-functioning difficulties.

### 2.3 Procedures and ethical considerations

For both samples, data collection was conducted collectively during scheduled in-person classroom sessions, with research personnel present to facilitate the administration. Secondary-school students completed the paper-and-pencil questionnaire with a research-team member present, whereas university students completed the same questionnaire through an electronic form with support from previously trained psychology students. In both cases, facilitators provided standardized instructions and addressed procedural questions without monitoring or influencing individual responses. Thus, the general setting, collective administration, procedural support, instrument content, and response options were comparable across samples, although the response medium differed.

All ethical procedures required for the study were followed in accordance with national regulations and the Declaration of Helsinki. The study received institutional approval from the Evaluation Committee of the Vice-Rectorate for Student Orientation and Affairs of the National Autonomous University of Honduras (VOAE official letter No. 383-2022). In addition, authorization was obtained from the participating secondary schools, as documented in the institutional approval letter signed by school authorities on March 10, 2023.

All participants provided voluntary informed consent before taking part in the study. The consent form described the study purpose, methodological procedures, participants’ rights, and the right to withdraw at any time. For participants under 18 years of age, parental or guardian informed consent and participant assent were obtained.

### 2.4 Data analysis

All analyses were conducted in R/RStudio (Posit Software, PBC, 2026; R Core Team, 2026). Confirmatory factor analysis (CFA) was performed with the *lavaan* package; descriptive statistics were obtained using *psych* and *skimr;* and network analyses were conducted with *bootnet*, *qgraph*, *mgm*, *igraph*, and *NetworkComparisonTest*.

In the first stage, descriptive statistics were computed for the eight executive-function domains assessed by the EFECO. Means, standard deviations, minimum and maximum values, skewness, and kurtosis were estimated. In addition, the mean score for each domain was expressed as a percentage of its maximum observed score, as a descriptive indicator of the relative intensity of self-reported executive-function difficulties across dimensions.

Before estimating the networks, the dimensional structure of the EFECO was evaluated through item-level confirmatory factor analysis (CFA). Given the ordinal nature of the response scale, the weighted least squares mean and variance adjusted estimator (WLSMV) was used. Model fit was evaluated using the comparative fit index (CFI), Tucker–Lewis index (TLI), root mean square error of approximation (RMSEA), and standardized root mean square residual (SRMR). Values of CFI and TLI > 0.95, RMSEA < 0.08, and SRMR < 0.08 were considered indicative of adequate fit (Hu and Bentler, 1999). Reliability was examined using omega coefficients (*ω*) and composite reliability (CR), which provide estimates of the proportion of reliable variance attributable to the common construct relative to measurement error (Raykov, 1997). These analyses supported the use of the eight EFECO dimensions as observed nodes in the network models. The results of these CFA and reliability analyses are reported in the instrument description section.

To determine whether the EFECO measured the same eight domains comparably in the secondary-school and university samples before interpreting between-sample differences in network structure, item-level measurement invariance was examined using multigroup confirmatory factor analysis (Putnick and Bornstein, 2016). Because the items were ordinal, the models were estimated using WLSMV with theta parameterization (Wu and Estabrook, 2016). Configural invariance assessed equivalence of the factor structure; metric (or weak) invariance tested equality of factor loadings; scalar invariance additionally tested equality of item thresholds; and strict invariance additionally tested equality of residual variances (Wu and Estabrook, 2016). CFI and RMSEA (90% CI) were reported, with SRMR considered complementary. Invariance was supported when ΔCFI ≤ 0.010 and ΔRMSEA ≤ 0.015; complementary ΔSRMR criteria were ≤ 0.030 for metric and ≤ 0.010 for scalar and strict invariance (Chen, 2007). Given the sensitivity of *χ*^2^-difference tests to sample size and their variable performance with ordinal WLSMV models, decisions were based primarily on changes in approximate fit indices, whereas robust model *χ*^2^ values were reported descriptively (Sass et al., 2014; Putnick and Bornstein, 2016).

The network analysis followed three sequential steps: network estimation, assessment of accuracy and stability, and a secondary exploratory between-sample comparison (Burger et al., 2020). The eight EFECO dimensions were entered as nodes and all retained participants had complete EFECO data. The global network was estimated as a *Gaussian graphical model (GGM),* where edges represent partial associations between executive-function domains after controlling for all remaining nodes (Epskamp and Fried, 2018; Isvoranu et al., 2022). Because the domain scores were not assumed to be normally distributed, Spearman correlations were used (Isvoranu and Epskamp, 2021). The network was estimated with the *ggmModSelect* procedure implemented in *bootnet*, using stepwise model selection and the extended Bayesian information criterion (EBIC) (Isvoranu and Epskamp, 2021; Isvoranu et al., 2022). The graphical layout was generated using the spring layout in *qgraph*, which follows a force-directed logic in which more strongly connected nodes are placed closer together (Fruchterman and Reingold, 1991).

Several network properties were examined. At the global level, density (D) was calculated as the proportion of non-zero edges relative to all possible edges, and transitivity (C^△^) was estimated to describe the tendency of nodes to form interconnected clusters (Isvoranu et al., 2022). At the node level, predictability was estimated with *mgm* and expressed as R^2^, indicating the proportion of variance in each node explained by its neighboring nodes (Haslbeck and Waldorp, 2018). Predictability was displayed graphically as rings around each node.

Centrality was examined using strength and expected influence (EI). Strength was used because it summarizes the overall magnitude of a node’s connections, whereas EI was included because it preserves the sign of associations and is therefore more informative when positive and negative edges may coexist (Bringmann et al., 2019). Other indices, such as closeness and betweenness, were not reported because their interpretation can be less stable and less suitable in psychological networks, especially when negative edges are present (Bringmann et al., 2019; Hallquist et al., 2021).

The accuracy of edge weights was evaluated using non-parametric bootstrapping with 1,000 resamples. This procedure was used to compare the edge estimates obtained in the original sample with the bootstrap mean estimates and to compute confidence intervals (CIs), with narrower intervals indicating greater precision (Epskamp, 2020). Edge inclusion plots were also inspected to identify how frequently each parameter was retained or set to zero across bootstrap samples.

The stability of centrality indices was examined using a case-dropping bootstrap with 1,000 resamples for strength and expected influence. This procedure progressively removes increasing proportions of cases and correlates the resulting centrality estimates with those obtained from the original sample. Stability was summarized using the correlation stability coefficient (CS), which indicates the maximum proportion of cases that can be dropped while retaining a correlation of at least 0.70 with the original centrality estimates. Following recommended criteria, CS values above 0.25 were considered the minimum acceptable level, whereas values above 0.50 were considered preferable for stable interpretation (Epskamp and Fried, 2018).

As a secondary exploratory analysis, networks were compared between the secondary-school and university samples. Separate networks were estimated for each sample using the same procedure as the full-sample network and plotted with a shared average layout. Between-sample differences in overall network structure, global strength, individual edge weights, node strength, and expected influence were tested using the Network Comparison Test (NCT) with 1,000 permutations (Van Borkulo et al., 2023). To account for multiple comparisons, the Holm–Bonferroni correction (HB) was applied separately to the 28 edge comparisons and the 16 node-centrality comparisons (Van Borkulo et al., 2023). Holm–Bonferroni-adjusted *p* values below 0.05 were considered statistically significant. As a complementary descriptive analysis, the Spearman correlation between corresponding edge weights was calculated, and absolute edge-weight differences were inspected to identify the largest between-sample discrepancies. This correlation was treated descriptively and not as a test of network equivalence.

## 3 Results

### 3.1 Preliminary analyses

Descriptive statistics for the eight EFECO domains are presented in Table 2. Because the domains differ in the number of items and maximum possible scores, mean scores were also expressed as percentages of their respective observed maximum values to facilitate comparisons of the relative intensity of self-reported executive-function difficulties across dimensions. Overall, relative intensity values were low to moderate, ranging from 19.57 to 33.00%. Emotional control showed the highest relative mean score (*M* = 6.93, SD = 4.65; 33.00%), followed by planning (*M* = 5.31, SD = 3.47; 26.55%) and flexibility (*M* = 4.21, SD = 2.61; 26.31%). In contrast, organization (*M* = 4.50, SD = 3.81; 19.57%) and monitoring (*M* = 5.23, SD = 3.33; 20.12%) showed the lowest relative scores. All domains showed positive skewness, indicating a concentration of scores toward the lower end of the scale. Kurtosis values were generally moderate, although monitoring showed the highest kurtosis value (2.50), suggesting a more peaked distribution for this domain.

**Table 2:** Descriptive statistics for the EFECO executive-function domains.

Dimension	Mean	SD	Median	Min.	Max.	Hist	Skewness	Kurtosis	%
Inhibition	6.44	4.02	6	0	28	▇▇▂▁	0.94	1.59	23.00
Flexibility	4.21	2.61	4	0	16	▇▇▂▁	0.79	1.02	26.31
Emotional control	6.93	4.65	6	0	21	▇▇▅▂	0.77	0.24	33.00
Monitoring	5.23	3.33	5	0	26	▇▅▁▁	1.18	2.50	20.12
Planning	5.31	3.47	5	0	20	▇▇▂▁	0.85	0.92	26.55
Organization	4.50	3.81	4	0	23	▇▅▁▁	1.19	1.84	19.57
Initiative	7.71	4.65	7	0	30	▇▇▂▁	0.87	1.29	25.71
Working memory	7.54	4.86	7	0	30	▇▇▂▁	0.97	1.40	25.13

SD, Standard deviation; Min., Minimum; Max., Maximum; Hist, Mini-histograms; %, Mean score expressed as a percentage of the maximum observed score for each executive-function dimension.

Table 3 presents the item-level measurement invariance results. The configural model supported the same eight-factor structure in both samples, and progressively constraining factor loadings, item thresholds, and residual variances produced no meaningful deterioration in model fit. Accordingly, configural, metric, scalar, and strict invariance were supported, providing evidence that the EFECO domains were measured comparably in the secondary-school and university samples.

**Table 3:** Measurement invariance of the EFECO across the secondary-school and university samples.

Model	*χ*^2^ (df)	CFI	ΔCFI	RMSEA [90% CI]	ΔRMSEA	SRMR	ΔSRMR
Configural	18,922.126 (4,232)	0.969	–	0.055 [0.054, 0.055]	–	0.067	–
Metric	22,514.462 (4,291)	0.962	0.**007**	0.060 [0.060, 0.061]	0.**005**	0.073	0.**006**
Scalar	19,478.562 (4,358)	0.966	0.**004**	0.055 [0.054, 0.055]	0.**005**	0.067	0.**006**
Strict	23,380.391 (4,425)	0.960	0.**006**	0.061 [0.060, 0.061]	0.**006**	0.073	0.**006**

*χ*^2^, chi-square; df, degrees of freedom; CFI, comparative fit index; RMSEA, root mean square error of approximation; CI, confidence interval; SRMR, standardized root mean square residual. Reported Δ values are absolute changes relative to the preceding model. Invariance criteria: |ΔCFI| ≤ 0.010 and ΔRMSEA ≤ 0.015; ΔSRMR was considered complementary (≤0.030 for metric; ≤0.010 for scalar and strict invariance). Bold values indicate changes in fit indices that met the prespecified criteria for measurement invariance.

### 3.2 Global network properties

The global network was highly connected. Of the 28 possible edges among the eight EFECO domains, 23 were distinct from zero, resulting in a density of 82.14%. The proportional edge density was 0.82, indicating that most executive-function domains were conditionally associated after controlling for the remaining nodes. The global transitivity coefficient was 0.82, suggesting a high tendency of the domains to form interconnected triads within the network.

### 3.3 Estimation of the network and centrality indices

Figure 1 shows the global network structure and centrality indices of the eight executive-function domains. Edge weights ranged from −0.08 to 0.32, with most associations being positive. The strongest partial associations were observed between inhibition and emotional control (0.32), planning and working memory (0.32), and initiative and working memory (0.30). Other relatively strong connections were observed between flexibility and emotional control (0.27), monitoring and initiative (0.25), and planning and organization (0.22). Two small negative edges were retained in the network: emotional control–planning (−0.08) and inhibition–initiative (−0.07).

**Figure 1:**
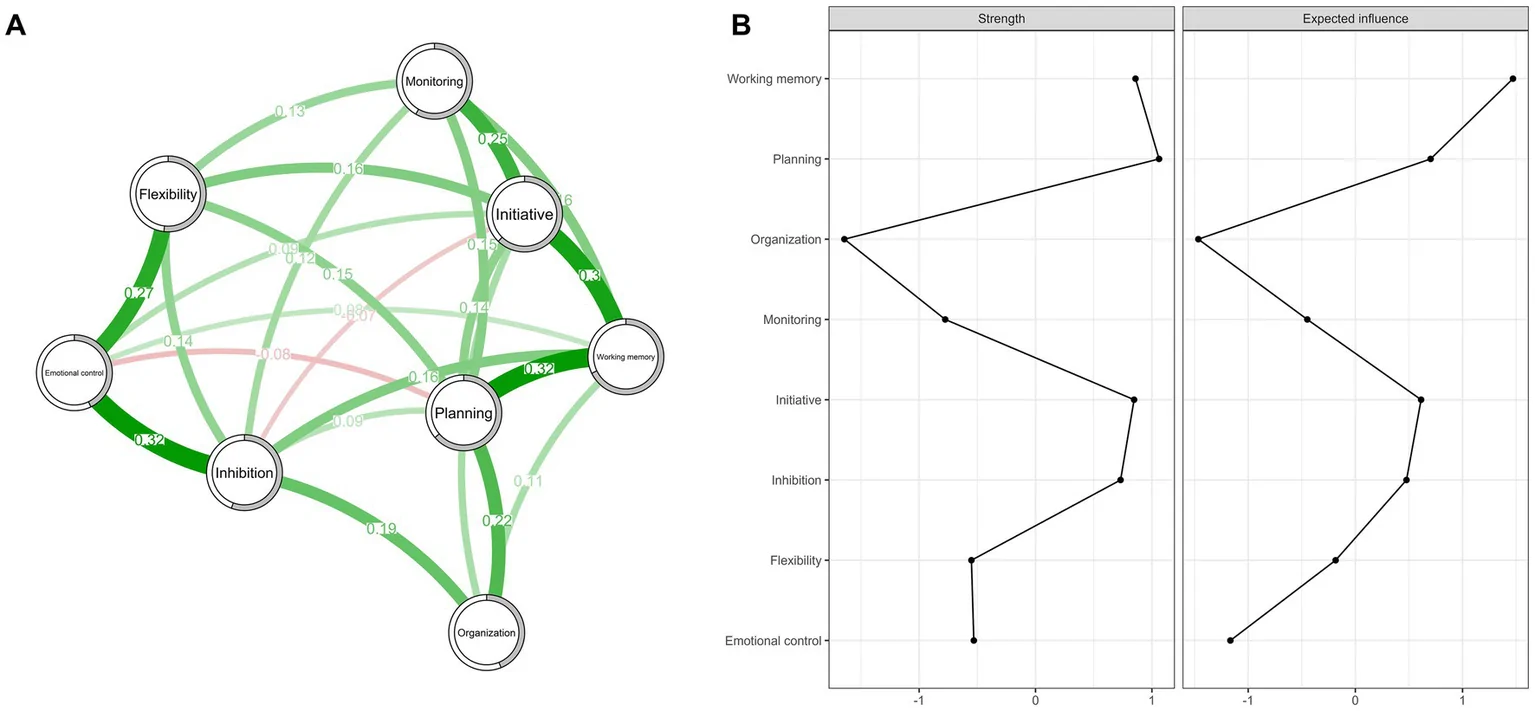
Global network structure and centrality of the EFECO executive-function domains. In **(A)** green and red edges indicate positive and negative partial associations, respectively; edge thickness reflects association strength, and rings indicate predictability (*R*²). **(B)** shows standardized centrality indices.

Predictability values indicated that a substantial proportion of variance in each domain was explained by its neighboring nodes. Node predictability ranged from *R*^2^ = 0.43 for emotional control to *R*^2^ = 0.68 for working memory. The highest predictability values were observed for working memory (*R*^2^ = 0.68), planning (*R*^2^ = 0.64), and initiative (*R*^2^ = 0.62), whereas the lowest values were observed for emotional control (*R*^2^ = 0.43) and organization (*R*^2^ = 0.44).

Regarding centrality, planning showed the highest standardized strength, followed by working memory and initiative. For expected influence, working memory showed the highest value, followed by planning and initiative. In contrast, organization showed the lowest values for both strength and expected influence.

### 3.4 Accuracy and stability of the global network

The accuracy and stability analyses are presented in Figure 2. In Panel A, the bootstrap mean estimates closely overlapped with the original sample estimates across most edges, suggesting that the estimated edge weights were generally precise. The confidence intervals were narrower for the strongest edges, particularly inhibition–emotional control, planning–working memory, initiative–working memory, flexibility–emotional control, and monitoring–initiative. Panel B showed that most retained edges were consistently included across bootstrap samples, although some weaker parameters showed a higher frequency of being set to zero.

**Figure 2:**
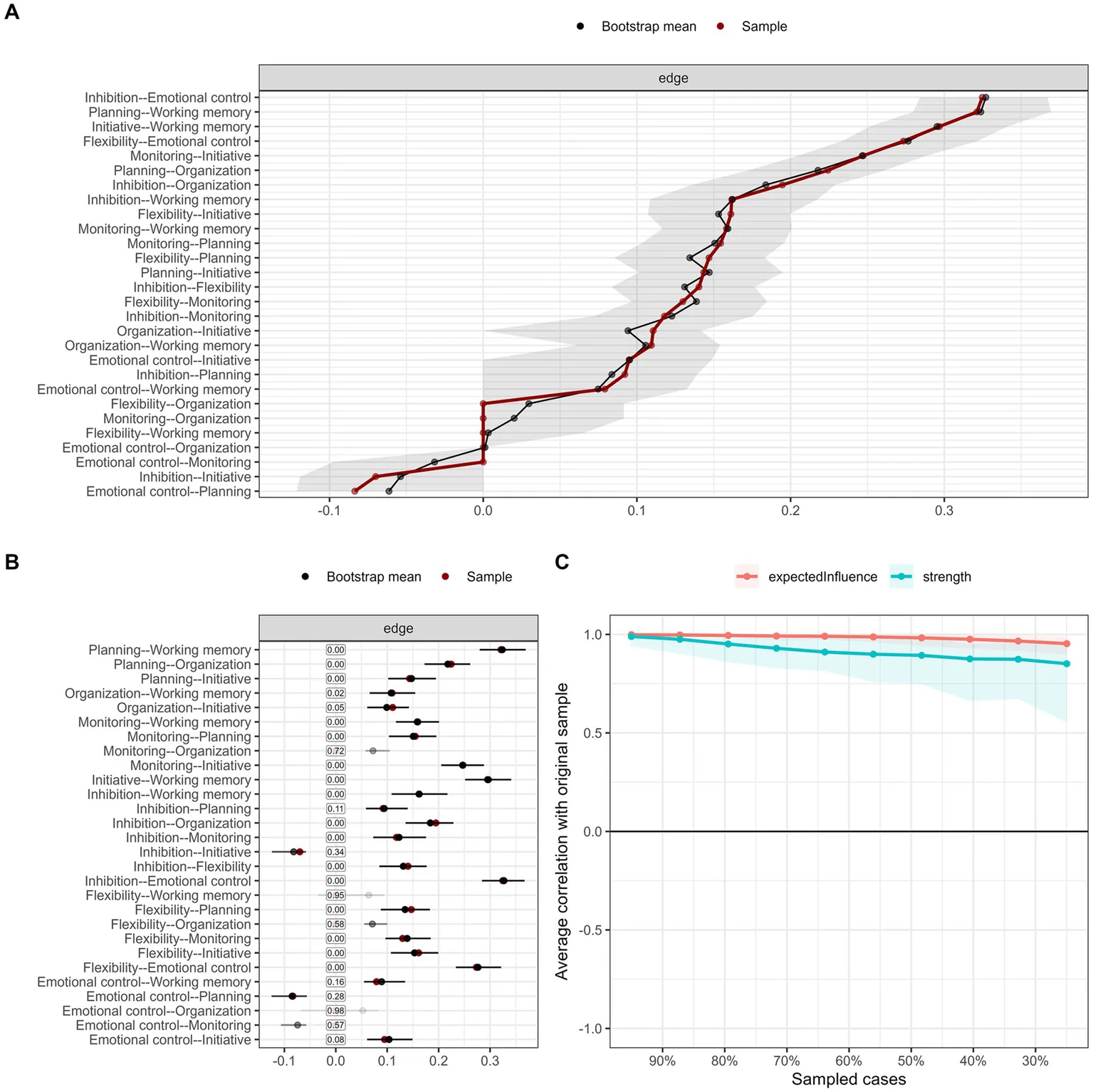
Accuracy and stability of the global EFECO network estimates. **(A)** Bootstrap confidence intervals for edge-weight accuracy. **(B)** Edge inclusion across bootstrap samples. **(C)** Case-dropping stability for strength and expected influence.

Regarding stability, the case-dropping bootstrap indicated high stability for both centrality indices. The CS coefficient was 0.75 for expected influence and 0.75 for strength, exceeding the preferred threshold of 0.50. As shown in Panel C, the average correlations between the original and resampled centrality estimates remained high even after progressively removing cases, supporting the stable interpretation of strength and expected influence.

### 3.5 Network comparison between the secondary-school and university samples

Figure 3 presents the networks estimated separately for secondary and higher education samples using a common layout. The exploratory Network Comparison Test did not detect a statistically significant difference in overall network structure between the samples (*M* = 0.15, *p* = 0.343). Global strength also did not differ significantly (*S* = 0.29, *p* = 0.253). As a complementary statistic, the corresponding edge weights were positively correlated (Spearman’s *ρ* = 0.71; bootstrap mean *ρ* = 0.69).

**Figure 3:**
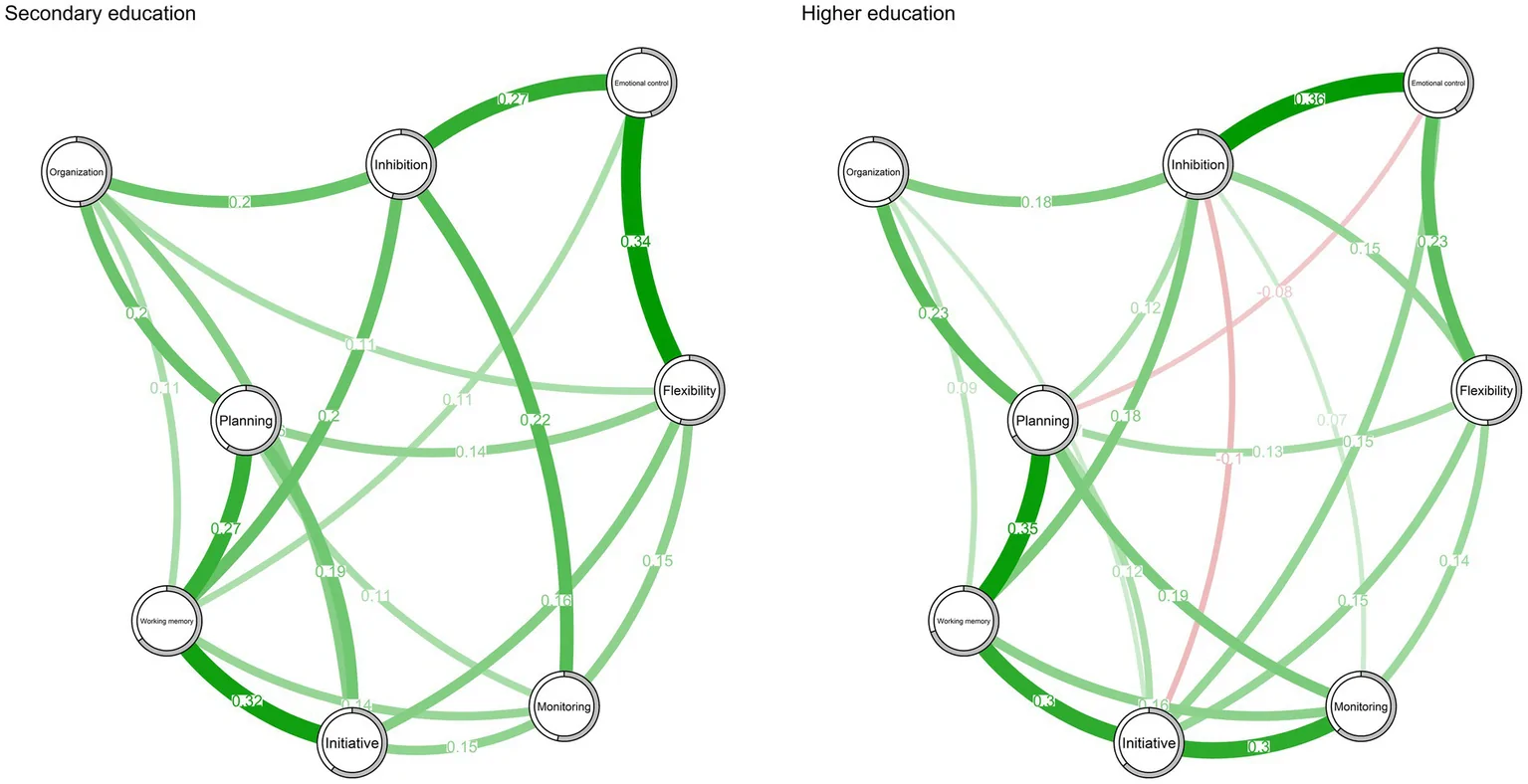
EFECO network structures in the secondary-school and university samples.

Despite the nonsignificant global tests, exploratory local comparisons yielded Holm–Bonferroni-adjusted *p* values below 0.05 for the following edges: inhibition–flexibility (*E* = 0.15, *p*_adj_ = 0.048), inhibition–emotional control (*E* = 0.09, *p*_adj_ = 0.047), flexibility–emotional control (*E* = 0.11, *p*_adj_ = 0.024), inhibition–monitoring (*E* = 0.15, *p*_adj_ = 0.026), flexibility–organization (*E* = 0.11, *p*_adj_ = 0.049), emotional control–initiative (*E* = 0.15, *p*_adj_ = 0.032), and monitoring–initiative (*E* = 0.15, *p*_adj_ = 0.002).

Figure 4 summarizes centrality indices and absolute edge-weight differences between the secondary-school and university samples. In Panel A, centrality comparisons identified differences in strength for inhibition (*p*_adj_ = 0.048), planning (*p*_adj_ = 0.008), and organization (*p*_adj_ = 0.003), and in expected influence for planning (*p*_adj_ = 0.020) and organization (*p*_adj_ = 0.002). Planning showed higher centrality in the university sample, whereas organization showed higher centrality in the secondary-school sample. Panel B displays the absolute differences between corresponding edge weights descriptively. The largest differences were observed for inhibition–flexibility, inhibition–monitoring, emotional control–initiative, and monitoring–initiative, all with absolute differences of 0.15.

**Figure 4:**
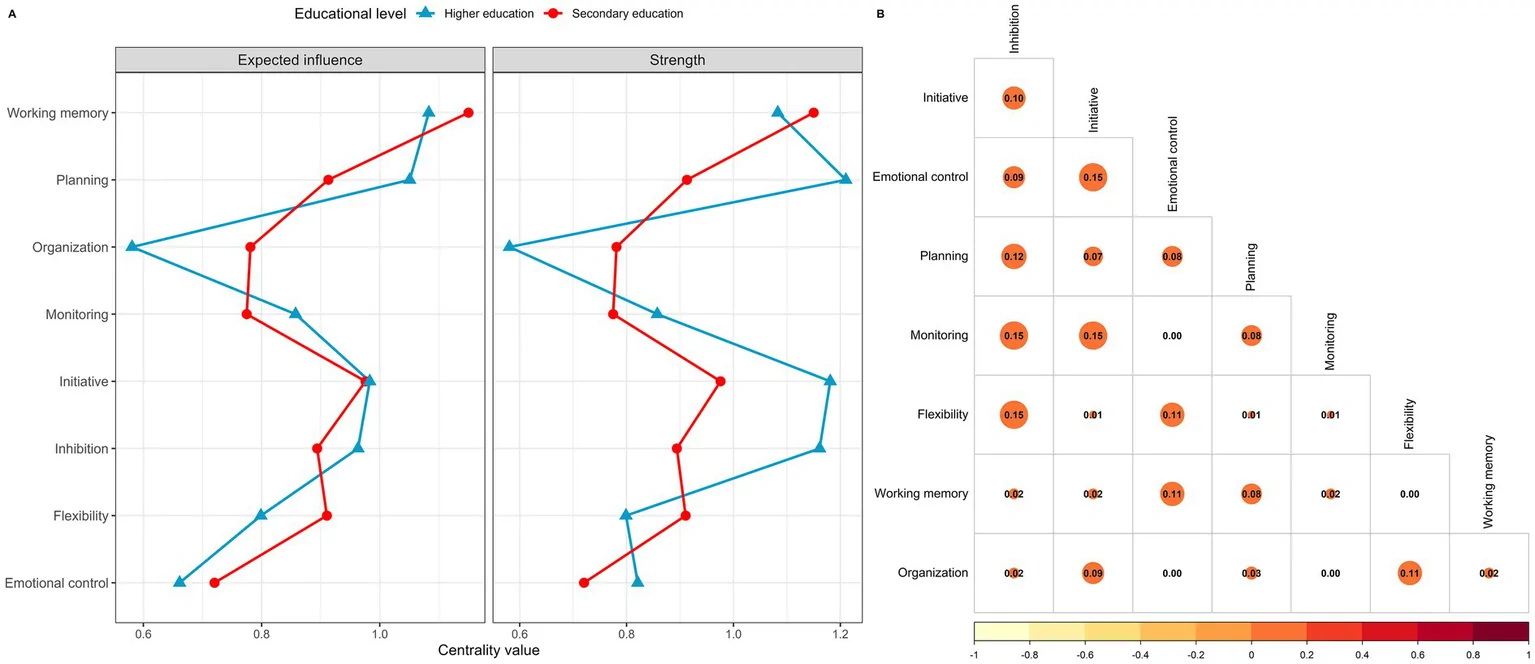
Centrality indices and absolute edge-weight differences between the secondary-school and university samples. **(A)** Centrality indices for the secondary-school and university samples. **(B)** Absolute differences between corresponding edge weights in the two sample networks. Inferential local comparisons were conducted using the Network Comparison Test with Holm–Bonferroni adjustment.

## 4 Discussion

This study primarily mapped the network structure of self-reported everyday executive-functioning difficulties in a large, combined sample of secondary-school and university students from Honduras. Two central findings emerged from the global network. First, the EFECO domain scores showed a dense pattern of conditional associations. Second, planning, working memory, and initiative occupied the most central positions in the network. As a secondary exploratory finding, the comparison between the independently recruited samples did not detect statistically significant differences in global network structure or global strength, although localized differences were observed in selected edges and centrality indices.

The global network was dense: 23 of 28 possible edges were retained, and global transitivity reached 0.82. This pattern indicates that most EFECO domain scores retained conditional associations after accounting for the remaining domains, but it does not by itself demonstrate that everyday executive difficulties operate as an interconnected psychological system. This analysis extends prior EF network evidence, based mainly on performance-based indicators (Karr et al., 2022; Menu et al., 2022, 2024; Younger et al., 2023), to an ecological self-report measure and provides geographically situated evidence from the participating secondary-school and university student samples in Honduras, a context not previously examined using this approach.

The level of interconnection observed here should be interpreted in light of the ecological, self-report nature of the EFECO. These findings reflect perceived everyday difficulties rather than standardized task performance, and rating scales and performance-based tasks are known to show limited convergence (Toplak et al., 2013). At a descriptive assessment level, the findings highlight the potential value of examining patterns across multiple EFECO domains rather than interpreting a single domain score in isolation. The educational relevance of this implication remains to be validated against external criteria.

Planning, working memory, and initiative had the highest centrality estimates in the global network. Together with their high predictability (*R*^2^ = 0.64, 0.68, and 0.62, respectively), these estimates indicate that their subscale scores were relatively more connected to the remaining EFECO domain scores and that neighboring domain scores accounted for a substantial proportion of their variance within the estimated network. Conceptually, this descriptive pattern can be situated within the literature on goal-directed academic behavior: planning helps organize future actions, working memory maintains task-relevant information, and initiative supports the beginning of activities without excessive external prompting (Dörrenbächer-Ulrich and Bregulla, 2024). More broadly, it can be considered alongside models of EF as a system of control processes supporting goal-directed regulation (Miyake et al., 2000; Diamond, 2013) and evidence showing that self-reported attention, planning, self-control, and self-monitoring are associated with study progress in first-year higher education students (Baars et al., 2015). This conceptual correspondence is informative and hypothesis-generating rather than evidence that these domains are functionally or causally more important or constitute intervention targets.

The strongest edges were consistent with two broad regulatory patterns. Associations among planning, working memory, initiative, and organization reflected a cognitive-organizational pattern related to structuring tasks, maintaining relevant information, and initiating academic activities. In turn, the inhibition–emotional control and flexibility–emotional control edges reflected an emotional-behavioral pattern in which difficulties controlling impulses, adapting to changing demands, and regulating emotional responses tended to co-occur. This pattern echoes the conduct-supervisory and cognition-supervisory dimensions previously proposed for the EFECO (Ramos-Galarza et al., 2019), providing convergent descriptive evidence from a network perspective. These associations should not be interpreted directionally, and the small negative edges retained in the network should be treated as descriptive features of the estimated conditional structure rather than as evidence of compensatory or antagonistic processes. Taken together, these centrality and edge patterns provide descriptive information about conditional connectivity and relative node positioning that is not directly represented by the correlated-factor structure of a confirmatory factor model, thereby complementing rather than replacing the latent-variable account of the EFECO. More broadly, the integration of item-level latent-variable analyses with domain-level network modeling provides complementary information about the measurement structure of the EFECO and the conditional relations among its executive-function domains.

The exploratory comparison between the secondary-school and university samples did not detect statistically significant differences in global network structure (*M* = 0.15, *p* = 0.343) or global strength (*S* = 0.29, *p* = 0.253). The moderate correlation between the corresponding adjacency matrices (*ρ* = 0.71) additionally indicated descriptive correspondence between the estimated networks. These findings indicate that global differences were not detected with the present data and analytic procedure; they do not establish network equivalence or confirm that the two samples share the same network structure.

Holm–Bonferroni-adjusted local comparisons identified several edge and centrality contrasts, including greater strength and expected influence for planning in the university sample and for organization in the secondary-school sample. Given the nonsignificant global tests, these local findings were interpreted as secondary and exploratory rather than as evidence of overall differences between the networks. One plausible, non-exclusive contextual interpretation is that planning may be more closely connected to other perceived executive difficulties under conditions involving greater autonomy, multiple deadlines, reduced external guidance, and independent regulation of academic work (Baars et al., 2015; Dörrenbächer-Ulrich et al., 2024). Conversely, organization may be more embedded in secondary students’ everyday functioning because materials, routines, and externally structured academic activities remain particularly salient. These interpretations remain tentative and should be regarded as contextual hypotheses rather than as effects attributable specifically to educational level.

These exploratory comparisons extend previous cross-sectional research contrasting secondary-school and university samples in related regulatory processes and performance-based executive functioning (Ben-Eliyahu and Linnenbrink-Garcia, 2015; Hederich-Martínez et al., 2018; Nederhand et al., 2021; Marquez-Ramos et al., 2023). As in that literature, the present design cannot disentangle age, educational experience, selection into higher education, or measurement characteristics from sample membership. Accordingly, the localized findings should be interpreted as sample-specific and hypothesis-generating rather than as evidence of developmental change or an effect attributable to educational level.

Because these localized contrasts were limited in magnitude and were identified despite nonsignificant global tests, they require independent replication. Future longitudinal studies following students across educational transitions are needed to examine intraindividual change. Matched cross-sectional studies with overlapping age ranges, more balanced sex distributions, multiple institutions, comparable sampling frames, and the same response medium would also help distinguish educational context from developmental, compositional, institutional, and procedural influences.

This study offers three main contributions. First, it provides a network-based description of conditional associations, relative centrality, and predictability across the eight executive-function domains assessed by the EFECO—information not directly provided by bivariate correlations or the correlated-factor structure. Second, it extends EF network research beyond performance-based tasks to self-reported everyday executive difficulties, while supporting item-level measurement invariance and examining the accuracy and stability of the estimated network. Third, with 2,330 participants, it provides geographically situated evidence from Honduras and an initial, explicitly exploratory account of global and localized patterns across independently recruited secondary-school and university samples. Together, these contributions establish a descriptive baseline and generate testable hypotheses for longitudinal, multimethod, and outcome-linked research.

These strengths should be considered alongside important limitations. The cross-sectional design and independently recruited samples preclude conclusions about developmental change, intraindividual trajectories, or effects of the transition from secondary to higher education. The samples differed in age and sex composition, while institutional setting, sampling frame, and administration format were aligned with sample membership. Consequently, observed between-sample contrasts cannot be attributed specifically to educational level and may reflect developmental, compositional, institutional, cohort, procedural, or other unmeasured differences. In addition, the non-probability purposive sampling strategy and recruitment from two secondary schools and one university campus limit the representativeness and generalizability of the findings beyond the participating institutions. Accordingly, the results should not be interpreted as nationally representative of Honduran secondary-school or university students.

Response medium was fully confounded with sample membership, so subtle mode-related effects on response patterns and network estimates cannot be independently identified or excluded. However, both groups completed the same questionnaire during collective in-person classroom sessions under standardized instructions and supervision. School-based studies have found generally comparable scores or prevalence estimates across paper-and-pencil and electronic administration for most outcomes (Van De Looij-Jansen and De Wilde, 2008; Colasante et al., 2019), consistent with meta-analytic evidence of cross-mode score comparability (Muehlhausen et al., 2015; Weigold et al., 2018). Item-level multigroup CFA also supported configural, metric, scalar, and strict invariance, indicating comparable factor structure, loadings, thresholds, and residual variances across samples. These findings reduce the likelihood that the network contrasts merely reflect differential measurement functioning but do not establish equivalence between administration modes.

Measurement limitations are also important. Because all nodes were aggregated domain scores derived from a single self-report instrument, the network may be affected by self-perception, response style, social desirability, shared response format, potential overlap in item content, and common method variance. These measurement characteristics may have contributed to the observed edge strengths and high network density, which therefore cannot be assumed to reflect only substantive interdependence among executive-function domains.

The study did not include performance-based tasks, informant reports, or external criteria such as academic achievement, self-regulated learning, adaptation, mental health, or dropout risk. As a result, the present network describes the internal organization of EFECO domains, but it does not establish how this configuration relates to other forms of executive-function assessment or to educational outcomes. Accordingly, the node-level predictability estimates represent the proportion of variance in each EFECO domain explained by its neighboring domains and should not be interpreted as evidence of prediction of external educational or clinical outcomes.

Finally, the findings should be interpreted in light of the limitations of psychological network models. Edges represent undirected partial associations, not causal pathways, and centrality should not be interpreted as direct evidence of causal importance or immediate intervention priority. Local between-sample contrasts, especially those of limited magnitude or with Holm–Bonferroni-adjusted *p* values close to conventional thresholds, require replication. Future studies should use longitudinal and multimethod designs to examine whether everyday executive-functioning networks change across educational transitions and whether their structure predicts academic regulation, achievement, adaptation, mental health, or educational trajectories. Replications in other Latin American and low- and middle-income contexts would also help determine whether the present findings generalize beyond the Honduran educational setting.

## 5 Conclusion

This study identified a dense network of conditional associations among self-reported EFECO domain scores in the participating Honduran student samples. Planning, working memory, and initiative had the highest centrality estimates, and the strongest associations reflected cognitive-organizational and emotional-behavioral regulatory patterns. After item-level measurement invariance across the secondary-school and university samples was established through the strict level, the secondary exploratory comparison did not detect statistically significant differences in global network structure or global strength, although localized sample-specific contrasts were observed. The nonsignificant global tests do not establish network equivalence, and the localized contrasts should not be attributed specifically to educational level or developmental change. By integrating an ecological self-report measure with network analysis in a Central American educational context, this study extends the literature on executive functioning beyond performance-based approaches and provides geographically situated evidence from an underrepresented setting.

## Data Availability

The raw data supporting the conclusions of this article will be made available by the authors, without undue reservation.
